# SO_2_ Removal from Flue Gas by Char-Supported Fe-Zn-Cu Sorbent

**DOI:** 10.3390/ma18020394

**Published:** 2025-01-16

**Authors:** Yueying Li, Chuan Na, Jinxiao Dou, Jianglong Yu

**Affiliations:** 1 Key Laboratory for Advanced Coal and Coking Technology of Liaoning Province, University of Science and Technology Liaoning, Anshan 114051, Chinajianglongyu@163.com (J.Y.); 2 Suzhou Industrial Park Monash Research Institute of Science and Technology, Southeast University-Monash University Joint Graduate School, Suzhou 215123, China

**Keywords:** flue gas desulfurization, activated char, breakthrough adsorption capacity, Fe-Cu-Zn sorbents

## Abstract

In this study, the mechanisms of SO_2_ adsorption on lignite char and char-supported Fe-Zn-Cu sorbent (FZC sorbent) were investigated. The FZC sorbent was prepared by the impregnation of metal components on raw coal followed by steam gasification. Flue gas desulfurization experiments were carried out on a fixed-bed reactor at 100–300 °C by using simulated flue gas containing SO_2_/O_2_/H_2_O balanced by N_2_. The flue gas composition was monitored by using an online flue gas analyzer. The solid samples before and after desulfurization were analyzed by using X-ray diffraction (XRD), Fourier Transform Infrared (FTIR) spectroscopy, Thermogravimetric Analysis–Mass Spectroscopy (TG-MS), and Brunauer–Emmett–Teller (BET) surface area analysis. The experimental results showed that both lignite char and the FZC sorbent can effectively adsorb SO_2_ under the present experimental conditions. The presence of O_2_ and H_2_O in the flue gas promoted the adsorption of SO_2_ on the FZC sorbent. The SO_2_ adsorption capacity of the FZC sorbent increased with the increase in the temperature up to 250 °C. When the temperature was further increased to 300 °C, the SO_2_ adsorption capacity of the sorbents decreased rapidly. Under optimum experimental conditions with a space velocity of 1500 h^−1^, a desulfurization temperature of 250 °C, and 5% (vol) O_2_ and 10% (vol) H_2_O in the flue gas, the sorbents exhibited the longest breakthrough time of 280 min and breakthrough SO_2_ adsorption capacity of about 2200 mg (SO_2_) per gram sorbent.

## 1. Introduction

Sulfur dioxide (SO_2_) is recognized as a significant contributor to atmospheric pollution, resulting in acid rain and the severe poisoning of air, water, and soil. Eliminating sulfur dioxide is crucial to mitigating air and aquatic pollution and enhancing environmental protection. Flue gas desulfurization (FGD) is an efficient method for diminishing SO_2_ emissions and has been extensively implemented to remove industrially produced SO_2_ [1,2,3,4]. Flue gas desulfurization technologies are primarily categorized into wet and dry procedures, depending on the type of sorbent and the removal environment. Traditional desulfurization methods, such as wet desulfurization, function at low temperatures and need substantial investment and elevated running expenses. Moreover, wet desulfurization methods necessitate wastewater treatment facilities. Conversely, dry desulfurization [5] can enhance sulfur removal efficiency and decrease capital expenditures. The desulfurization agent is crucial in the dry desulfurization process.

Carbon materials and metal oxides have been employed for dry desulfurization [6,7]. Carbon materials, including activated carbon fibers (ACFs), activated carbons (ACs), and activated chars, are applicable for the low-temperature extraction of SO_2_ from flue gas. Owing to their physical and chemical properties, these materials exhibit significant adsorption capability for diverse gases [8,9]. Several investigations have been documented about the desulfurization properties of various carbon materials [10]. Sorbents infused with metal oxides as the active component require an initial temperature of over 350 °C for effective desulfurization [11]. Activated carbon (char) demonstrated a greater adsorption ability for sulfur at approximately room temperature. As the temperature rises, the sulfur capacity diminishes significantly [12]. The optimal method for flue gas desulfurization occurs within the exhaust temperature range of 120 to 250 °C. The elevated specific surface area, advanced microporous architecture, and excellent catalytic reactivity of activated char, combined with the robust sulfur retention capacity of metal oxides at elevated temperatures, render activated char-supported metal oxides highly promising for the desulfurization of flue gas at moderate temperatures [13]. Lignite is a plentiful and inexpensive form of coal, constituting about fifty percent of the world’s coal reserves. The elevated reactivity of lignite char renders it an advantageous medium for active metal oxides [14,15,16,17,18].

Tseng et al. [19] investigated the mechanisms of SO_2_ adsorption and the regeneration of activated carbon-supported copper oxide sorbents/catalysts within a fixed-bed reactor. Their findings indicated that SO_2_ was catalytically oxidized to SO_3_ over a copper phase in the presence of gaseous oxygen. It reacted with a copper active site to produce a sulfate bonded to copper without desorption into the gas phase. The activated carbon support did not participate in the sulfidation processes. The deactivated sorbent can be regenerated through direct thermal treatment in an inert atmosphere following SO_2_ adsorption, whereas carbon can reduce CuSO_4_ to Cu via its surface oxygen complexes. Copper is swiftly oxidized by oxygen in flue gas during the adsorption process. Flores et al. [20] investigated the influence of support type, composition, and reaction temperature on the reduction of SOx from flue gases utilizing CuO as the sorbent. The wet impregnation process synthesized CuO supported on alumina, titanium, and zirconium. The results indicated a direct association between the sorbents’ adsorption capacity, the support’s surface area, and CuO content. The adsorption capacity and desulfurization efficiency of the sorbents improved with the rise in temperatures.

The desulfurization characteristics of activated carbon and activated carbon-supported single or binary metal oxide sorbents are documented in the literature [21,22]. Nonetheless, limited studies have been conducted on the desulfurization properties of lignite char, which is crucial for the value-added and effective usage of this plentiful coal resource. Furthermore, there needs to be more reporting on the ternary composite sorbent for flue gas purification. This study synthesized the lignite char-supported Fe-Zn-Cu sorbent (FZC), and its desulfurization performance was systematically examined in a fixed-bed reactor. To enhance the comprehension of the essential mechanisms behind SO_2_ removal by the char-supported Fe-Zn-Cu sorbent, the desulfurization performances of lignite char and the FZC sorbent were examined. The influence of O_2_ and H_2_O concentrations in flue gas on desulfurization was examined [23,24]. Efforts were undertaken to examine potential reactions occurring in FZC desulfurization procedures. The newly prepared sulfidated sorbents were evaluated by using XRD, FTIR, TG-MS, and BET studies.

## 2. Materials and Methods

### 2.1. Sorbent Preparation

Shenhua lignite from the Inner Mongolia region of China was utilized to manufacture sorbents. The ultimate and proximate analyses of the lignite sample are displayed in Table 1. The coal sample was pulverized and classified into a 75–125 μm particle size range. The air-dried lignite sample was subjected to a 4 mol/L hydrochloric acid aqueous solution and agitated for 12 h. The slurry was further filtered and rinsed with deionized water until the pH of the filtrates stabilized between 5.0 and 5.5. By using an ultrasonic-assisted co-precipitation technique, the lignite sample was impregnated with an aqueous solution of the precursors (ferric nitrate, zinc nitrate, and copper nitrate). The weight ratio of iron to acid-washed coal was 1:10. Fe(NO_3_)_3_, Zn(NO_3_)_2_, and Cu(NO_3_)_2_ (Shenyang, China) were solubilized in deionized water with molar ratios of Fe:Zn: Cu of 2:1:1. The acid-washed coal was combined with ferric nitrate, zinc nitrate, and copper nitrate solutions by using an ultrasonic-assisted impregnation method for 5 h at 50 °C. Concentrated aqueous ammonia was added at one-minute intervals to the mixture until the pH of the solution reached 10. Ultrasound irradiation was maintained for one hour at 50 °C. The slurry was filtered and dried in a vacuum oven at 105 °C for 12 h. The dried sample underwent pyrolysis in nitrogen for 15 min and gasification in steam (15% vol. balanced with nitrogen) for 30 min in a fluidized-bed quartz reactor at approximately 727 °C to produce the FZC sorbent [25,26].

### 2.2. Desulfurization Experiments

**Figure 1:**
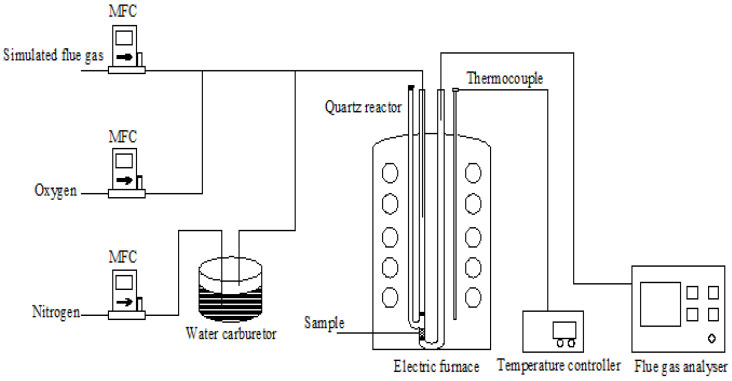
Schematic diagram of experimental setup.

The desulfurization tests of flue gas using the FZC were conducted in a fixed-bed quartz reactor with a 10 mm inner diameter, heated in an electric furnace. Mass flow controls delivered the simulated flue gas into the reactor, while steam was supplied through a water bath. The steam content was regulated by modifying the temperature of the water bath. An online gas analyzer (MRU-5) was employed to assess the concentration of SO_2_ in the exhaust gas. N_2_ served as the balancing gas in the trials. The desulfurization studies were conducted at a volumetric hourly space velocity (VHSV) of 1500 h^−1^ and within a temperature range of 100 to 300 °C. Approximately 1.3 g (2.4 mL) of sorbent was positioned at the center of the reactor. Before each test, the sorbent-containing reactor was heated to the specified temperature under nitrogen to remove air from the reaction environment. The constituents of the simulated gases and experimental circumstances are detailed in Table 2. Figure 1 illustrates the schematic diagram of the experimental setup.

### 2.3. Sorbent Characterization

The crystal structure and content of fresh and sulfidated sorbents were examined by using X-ray diffraction (XRD) with a X-ray diffractometer-7000 (Shimadzu, Kyoto, Japan). The X-ray diffraction pattern of the sample was obtained by using a step-scanning technique within the range of 2θ = 10–90° at a rate of 0.0033°/min. The metal oxide phases were identified by using Fourier Transform Infrared (FTIR) spectroscopy with a Nicolet IS5 mid-FT-IR spectrometer (Thermo Fisher, Waltham, MA, USA) [25], operating within the 4000–400 cm^−1^ range. The thermal decomposition of the samples was analyzed by using TG-MS (NETZSCH STA 449 F3 linked with NETZSCH QMS 403 C, NETZSCH-Gerätebau GmbH, Selb, Germany) in non-isothermal mode. Approximately 20 mg of sample with a 75–125 μm particle size was placed into an alumina crucible. The mass loss of the samples and the evolution of gaseous products were recorded concurrently. Samples were heated within the temperature range of 303–1473 K at a rate of 10 K·min^−1^ under a flow of argon gas at 30 mL·min^−1^.

## 3. Results and Discussion

### 3.1. Effects of Oxygen and Steam During Desulfurization Process

**Figure 2:**
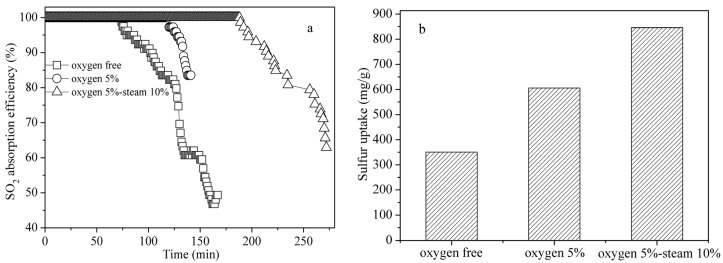
(**a**) SO_2_ breakthrough curve of FZC in different atmospheres; (**b**) breakthrough sulfur capacity of FZC in different atmospheres.

Figure 2a illustrates the impacts of the oxygen and steam in the flue gas on the removal of SO_2_ by the FZC sorbent. When 5% oxygen was present in the flue gas, the breakthrough time of the FZC was approximately 124 min, 48 min longer than when oxygen was absent. Figure 2b illustrates that in the presence of 5% oxygen in the flue gas, the breakthrough sulfur capacity of the sorbent attained was around 600 mg/g. This amount exceeded the scenario without oxygen by 250 mg/g. The results validated the beneficial impact of oxygen in the flue gas on desulfurization using the FZC sorbent. In the absence of oxygen alone, the oxygen functional groups on the sorbent surface supply oxygen to facilitate the oxidation of SO_2_. When flue gas contains oxygen, O_2_ will adsorb onto the char surface. The adsorbed O_2_ will then dissolve and convert into lattice oxygen, augmenting the oxidation of SO_2_. The absorbed oxygen interacts with metal oxides and SO_2_ to facilitate the catalytic oxidation of SO_2_ (Equations (5), (6) and (9)), hence improving SO_2_ elimination. Certain metal sulfides and metal sulfates were produced during the catalytic oxidation of SO_2_, which will be addressed in the XRD analysis section.

Figure 2a indicates that when the flue gas comprised 10% steam and 5% oxygen, the breakthrough time of the FZC sorbent was approximately 194 min. This figure exceeded the scenario with only 5% oxygen in the flue gas by 70 min. The enhanced sulfur capacity of the sorbent in the presence of 10% steam in the feed gas was approximately 270 mg/g greater than that of the steam-free flue gas, achieving a total of around 870 mg/g. The results indicated that steam presence is advantageous in the desulfurization process. This results from the interaction of SO_3_ with H_2_O to form H_2_SO_4_. The desorbed H_2_SO_4_ is subsequently maintained in the pores, augmenting active site recovery [10]. Oxygen and steam in flue gas from power plants indicate that the char-supported Fe-Zn-Cu sorbent is highly promising for industrial desulfurization applications.

**Figure 3:**
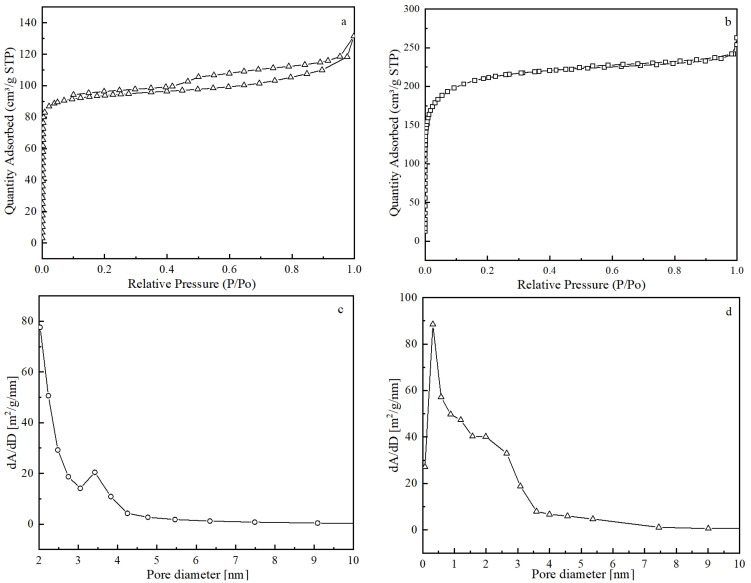
(**a**) Lignite char adsorption isotherms; (**b**) FZC adsorption isotherms; (**c**) lignite char pore size distribution curves; (**d**) FZC pore size distribution curves.

The SO_2_ adsorption efficiency of lignite char and char-supported FZC sorbent in the presence of O_2_ and H_2_O was examined at various desulfurization temperatures, with the findings being illustrated in Figure 3. It is evident that as the temperature rises, the desulfurization efficacy of lignite char diminishes. SO_2_ is eliminated by char primarily via physical adsorption and partially through chemical adsorption in the forms of SO_3_ and H_2_SO_4_. Elevated temperatures adversely impacted physical adsorption. As the temperature rose, the feed gas volume expanded, adversely affecting SO_2_ adsorption on the char surface. The restricted number of active sites on lignite char led to a reduction in desulfurization efficiency. Numerous investigations regarding the SO_2_ adsorption mechanism on activated carbon have been documented in the literature [27,28]. Zheng et al. investigated the elimination of SO_2_ on activated semi-coke and proposed that physically adsorbed SO_2_ can convert into chemically adsorbed SO_2_, a process contingent upon the reaction temperature. A reactive intermediate, the C(O) complex, is thought to be the primary active site in the oxidation of SO_2_, formed by the dissociative chemisorption of O_2_ onto the surface of activated semi-coke. The oxidation of SO_2_ to SO_3_ is crucial for the elimination of SO_2_. Figure 3 illustrates that the FZC sorbent exhibited markedly superior desulfurization efficacy compared with lignite char. This was ascribed to the disparity in structural characteristics between lignite char and FZC and the existence of active components on the FZC sorbent.

The BET adsorption and desorption curves are shown in Figure 3. It can be seen that lignite char is an H4-type hysteresis loop of the IV isotherm, indicating that lignite char is dominated by a mixed structure of micropores and mesopores, and the FZC adsorbent is a type I isotherm, which indicates that the FZC adsorbent is a microporous structure. The pore size distribution curve shows that the pore size of lignite is mainly distributed in the range of 2–3.5 nm and the pore size of FZC adsorbent is mainly distributed in the range of 1–2 nm. The pore structure parameters of these two materials were analyzed, and the results are shown in Table 3.

Table 3 presents the BET surface area, pore volume, and pore size of lignite char and FZC sorbent. The BET surface area of the FZC sorbent was nearly six-fold greater than that of char. The FZC exhibited a greater pore volume and a reduced average pore size compared with lignite char [29]. A substantial surface area signifies that numerous active sites can be accessible to the flue gas. FZC can offer additional active sites for SO_2_ adsorption, while the included Fe, Zn, and Cu oxides can supply lattice oxygen (O_2_−) to facilitate the oxidation of SO_2_ to SO_3_. The incorporation of Fe-Zn-Cu oxides markedly improved the desulfurization efficacy of char.

**Figure 4:**
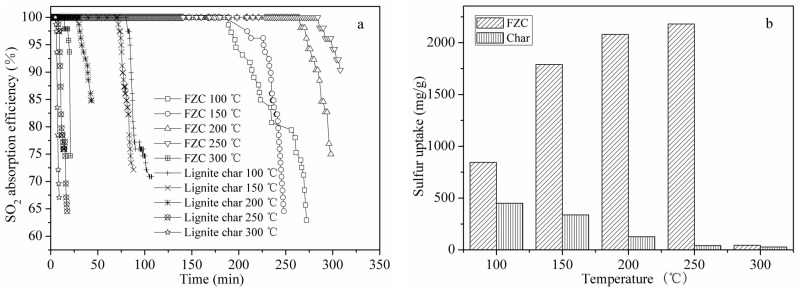
(**a**) SO_2_ breakthrough curve of lignite char and FZC sorbent at different temperatures; (**b**) breakthrough sulfur capacity of char and FZC sorbent at different temperatures. Experimental conditions: 2000 ppm SO_2_/N_2_/O_2_/H_2_O, 1500 h^−1^, and 100–300 °C.

Figure 4 indicates that the adsorption of SO_2_ on FZC escalated with the rise in temperatures, reaching up to 250 °C. Raising the temperature to 300 °C markedly reduced the SO_2_ removal efficiency of the sorbent. Two distinct methods in the flue gas desulfurization process utilizing char and FZC sorbent can be proposed. One involves the catalytic oxidation of SO_2_, while the other pertains to the interactions between metal oxides and SO_3_ during desulfurization. The catalytic oxidation of SO_2_ constitutes the rate-limiting stage in the desulfurization process. Elevated temperatures augmented the catalytic oxidation of SO_2_, facilitating desulfurization as the temperature increased. Nonetheless, above 300 °C, the desulfurization efficacy of the sorbents diminished significantly. A significant quantity of SO_3_ was produced at 300 °C [30]. The competing interaction of SO_3_ and SO_2_ with the active sites of the sorbent inhibited the adsorption of SO_2_, resulting in a reduction in the SO_2_ removal efficiency of the sorbent.

Figure 4b illustrates the sulfur absorption capacity of lignite char and FZC sorbent within the temperature range of 100–300 °C in the presence of 5% oxygen and 10% steam in the flue gas. The FZC sorbent had a much greater sulfur capacity than lignite char across all measured temperatures. Raising the temperature to 250 °C enhanced the sulfur absorption capacity of the FZC sorbent. The sulfur absorption capacity of the FZC rose from 750 mg/g at 100 °C to 2200 mg/g at 250 °C. Raising the temperature to 300 °C led to a substantial reduction in the sulfur capacity of the FZC sorbent. The sulfur capacity of lignite char exhibited an inverse correlation with desulfurization temperature. The sulfur capacity of lignite char diminished consistently as the temperature rose to 300 °C.

### 3.2. FTIR Analysis of Sorbents

**Figure 5:**
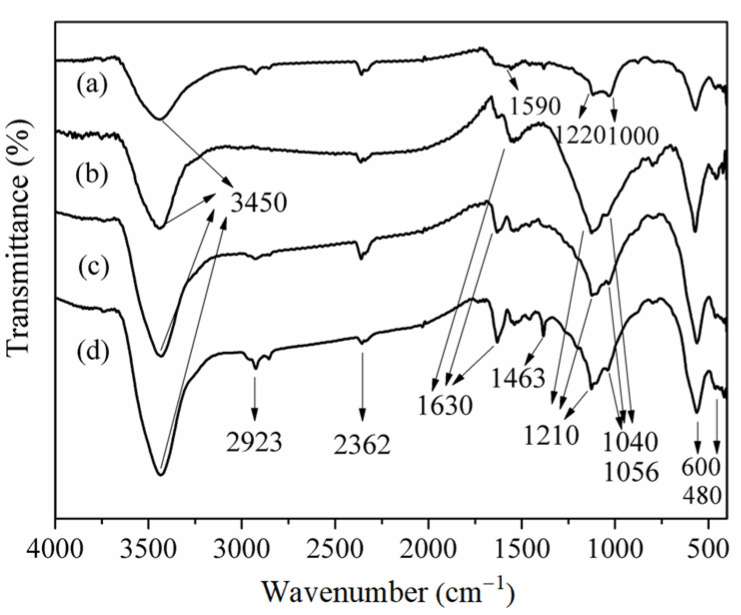
The FTIR spectra of (a) fresh sorbent, (b) sulfidated sorbent in 100 °C/oxygen-free/steam-free environment, (c) sulfidated sorbent in 100 °C/5% oxygen/steam-free environment, and (d) sulfidated sorbent in 100 °C/5% oxygen/10% steam environment.

The FTIR spectra of fresh and sulfidated sorbents under different experimental conditions were compared to investigate the changes in functional groups and the nature of the sulfur species. The FTIR spectra of fresh and sulfidated sorbents in different environments are shown in Figure 5. The assignment of bands in the IR spectra was obtained from the literature and is summarized in Table 4 [31].

A broad, strong peak at about 3450 cm^−1^, indicative of O–H stretching vibrations from hydroxyl groups and physically adsorbed water, was detected in all samples [32]. The band at 2923 cm^−1^ was ascribed to the υ(C–H) stretching vibration of methylene groups [33,34]. The band at 2362 cm^−1^ corresponds to the υ(C≡C) vibration in the alkyne functional group. Significant alterations in the IR spectra before and after desulfurization were noted in the 1700–400 cm^−1^ range. In the fresh sorbent (Figure 4a), the bands at 1220 and 1100 cm^−1^ are attributed to the C–O in ethers, while the band at 1590 cm^−1^ is associated with C=O (carboxyl-carbonates). The oxygen functional groups on the sorbent serve as active sites for the adsorption of SO_2_ and function as catalysts for the reaction between metal oxides and sulfur dioxide. The bands between 600 and 480 cm^−1^ were attributed to the adsorption of metal oxides [35,36,37] involved in the desulfurization reactions. In the deactivated sorbent in various experimental settings (b, c, and d in Figure 4), the band at 1056 cm^−1^ is attributed to the symmetric stretching vibration of –SO_3_ [38]. This band’s existence verifies the chemical adsorption of SO_2_ during the desulfurization process. The bands at 1210 and 1040 cm^−1^ were attributed to the adsorption of SO_4_^2−^ [39]. The bands at 600 and 480 cm^−1^ correspond to metal sulfates and sulfides formed during sulfur dioxide adsorption [40,41]. The examination of the IR spectra of both fresh and sulfidated sorbents revealed that the oxygen functional groups and metal oxides served as the active sites on the sorbent [42]. The data also validated the creation of H_2_SO_4_, metal sulfides, and metal sulfates during desulfurization [43].

### 3.3. XRD Analysis of Changes in Sorbents During Desulfurization

**Figure 6:**
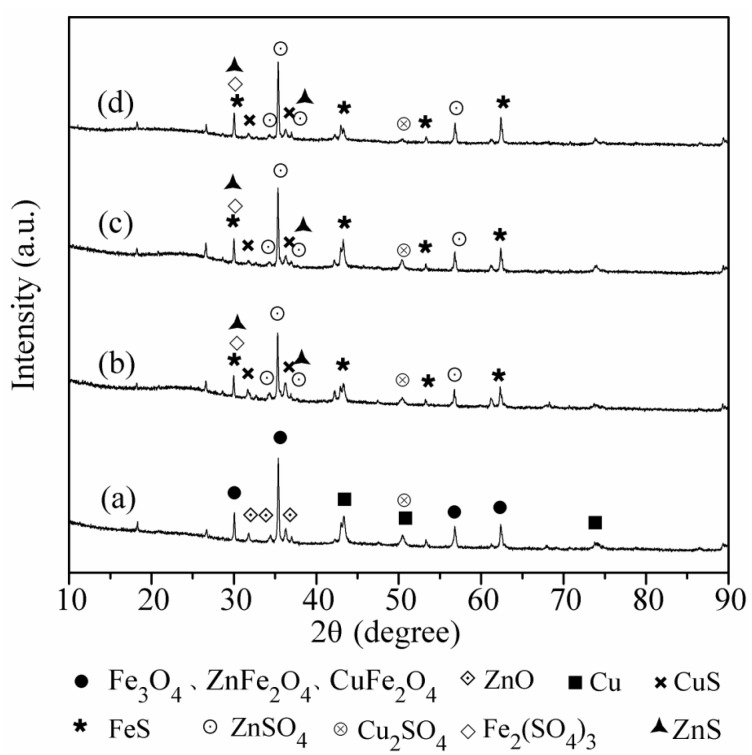
The XRD spectra of (a) fresh sorbent, (b) sulfidated sorbent in 100 °C/oxygen-free/steam-free environment, (c) sulfidated sorbent in 100 °C/5% oxygen/steam-free environment, and (d) sulfidated sorbent in 100 °C/5% oxygen/10% steam environment.

XRD analysis was employed to examine the crystal structure of fresh and sulfidated sorbents under different atmospheres, aiming to assess the influence of oxygen and steam in feed flue gas on SO_2_ removal. The findings of the XRD analysis are presented in Figure 5. Certain metal compounds were challenging to identify in the XRD pattern due to excessive dispersion and the formation of tiny crystals on the sorbent surface. Zinc ferrite (ZnFe_2_O_4_), copper ferrite (CuFe_2_O_4_), Fe_3_O_4_, ZnO, and Cu were the reactive species present on the newly prepared sorbent (Figure 6a). In the absence of oxygen, the intensity of peaks for reactive species in the sulfidated sorbents diminished, and a minor quantity of metal sulfides and metal sulfates was identified as the desulfurization result. Metal sulfates and metal sulfides were produced by the interaction of metal oxides with elemental metals and SO_3_. The elemental metals were produced by reducing metal oxides in the presence of char [19]. Metal sulfides may also form from the breakdown of metal sulfate in the presence of activated carbon in a reducing environment [44]. Carbon monoxide was detected throughout the desulfurization process, confirming the breakdown of metal sulfate. The specifics of chemical processes are provided in Equation (10). Metal sulfide may be regenerated more easily than metal sulfate, which is advantageous for the multiple-cycle application of sorbents [23,45].

In the presence of 5% oxygen in the input gas (Figure 6c), the intensity of the active components diminished significantly. Metal sulfides and metal sulfates were identified in the sulfidated sorbent. Figure 6d displays the XRD patterns of the sulfidated sorbent when the feed gas contained 10% steam and 5% oxygen. The intensity of peaks associated with desulfurization products increased, indicating that steam in flue gas can boost SO_2_ removal.

### 3.4. TG-MS Analysis of FZC Sorbents

**Figure 7:**
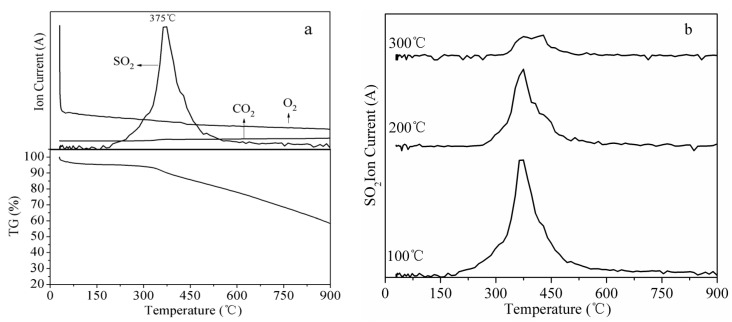
(**a**) TG and gas evolution curves during the thermal decomposition of sulfidated sorbents in oxygen-free flue gas; (**b**) SO_2_ evolution curves during the thermal decomposition of sulfidated sorbents at different temperatures. Experimental conditions: 2000 ppm SO_2_/N_2_, 100–300 °C, and 1500 h^−1^.

To better understand the mechanism of SO_2_ oxidation–adsorption, the sulfidated FZC sorbents were subjected to heat treatment by using TG-MS analysis. Figure 7a shows the weight loss and gas evolution curves during the thermal decomposition of the sulfidated sorbent at 100 °C. A slight weight loss was observed at around 50–150 °C and was attributed to moisture loss from the sulfidated sorbent. The primary weight loss was detected in a temperature range of 250–900 °C, corresponding to the evolution of SO_2_, the decomposition of sulfur species, and the evolution of CO_2_. The primary weight loss in the sorbent was attributed to the evolution of SO_2_, formed due to the decomposition of sulfur species produced during desulfurization. The evolution of SO_2_ from the sorbent started at around 250 °C and reached its maximum at 375 °C. Similar results have been reported in the literature [46,47,48,49]. No SO_2_ evolution was observed at temperatures higher than 600 °C. The decomposition of sulfur species and evolution of SO_2_ can be summarized in the following equations:

C–SO_3_ → SO_2_ +C (O) (1)

C–SO_3_ + C (O) → C + SO_2_ +CO_2_ (2)

MSO_4_ → MO_X_ + SO_2_ + O_2_ (3)

MS + O_2_→MO_X_ + SO_2_ (4)

2H_2_SO_4_ + C →2SO_2_ + CO_2_ + 2H_2_O (5)

MO_X_: metal oxide; MSO_4_: metal sulfate; MS: metal sulfide; C (O): a reaction intermediate from the desorption of C–SO_3_.

In the temperature range of 250–900 °C, a relatively constant evolution of CO_2_ was observed. CO_2_ may be formed during the decomposition of carboxylic acid functional groups, carboxylic anhydrides, and lactones of the char support and from the dissociated chemisorption of SO_2_ and the decomposition of H_2_SO_4_ [50]. Figure 6b shows the SO_2_ evolution curves during the thermal decomposition of sulfidated sorbents at different desulfurization temperatures when no oxygen and steam were present in the flue gas. The SO_2_ evolution increased with the decrease in the temperature in the absence of oxygen and steam.

**Figure 8:**
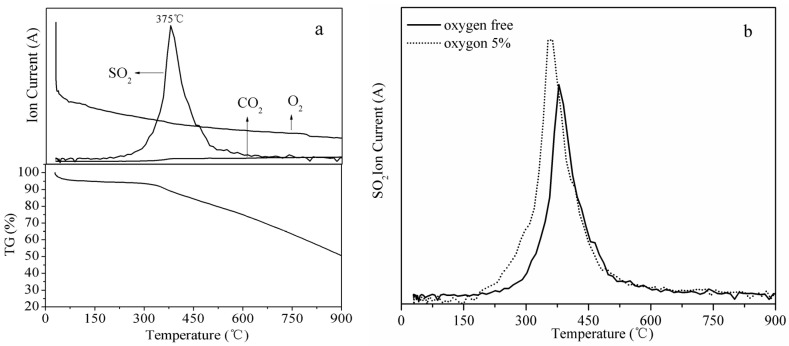
(**a**) TG and gas evolution curves during the thermal decomposition of sulfidated sorbents with 5% oxygen in the flue gas; (**b**) SO_2_ evolution curves during the thermal decomposition of sulfidated sorbents under oxygen-free conditions and with 5% oxygen in feed gas. Experimental conditions: 2000 ppm SO_2_/N_2_ or 2000 ppm SO_2_/N_2_/O_2_ 5%, 100 °C, and 1500 h^−1^.

Figure 8a shows the TG and main gas evolution curves during the thermal decomposition of the sulfidated sorbent at 100 °C when 5% oxygen was present in the feed gas. The positions of the peaks were similar to the curves of the sulfidated sorbent in an oxygen-free environment (Figure 6a). However, there was a slight difference in peak shape. Figure 8b shows that the evolution of SO_2_ was higher when 5% oxygen was present in the flue gas compared with the oxygen-free feed gas. These results indicate that the presence of oxygen in the flue gas enhanced SO_2_ removal. These results are consistent with the XRD analysis results and breakthrough sulfur capacity analysis.

**Figure 9:**
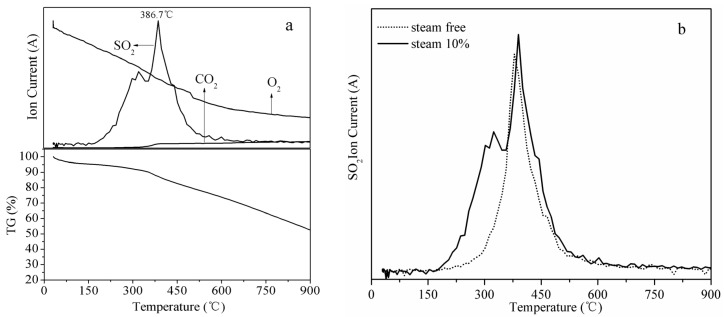
(**a**) TG and gas evolution curves during the thermal decomposition of the sulfidated sorbent with 10% steam in the flue gas; (**b**) SO_2_ evolution curves during the thermal decomposition of sulfidated sorbents in water-free or 10% steam flue gas environments. Experimental conditions: 2000 ppm SO_2_/N_2_ O_2_ 5% or 2000 ppm SO_2_/N_2_/O_2_ 5%/H_2_O 10%, 100 °C, and 1500 h^−1^.

Figure 9a shows the TG and evolution curves of the leading gases during the thermal decomposition of the sulfidated sorbent at 100 °C when 10% steam and 5% oxygen were present in the feed gas. The peak of the SO_2_ evolution curve occurred at a higher temperature compared with the other conditions tested (Figure 7a and Figure 8a). Figure 9b shows that the amount of SO_2_ released from the sulfidated sorbent when 10% steam was present in the flue gas was higher than when no steam was added to the feed gas. This result is also in good agreement with the desulfurization experiments discussed above.

The general mechanism of SO_2_ removal by the FZC sorbent based on desulfurization curves and XRD, FTIR, and TG-MS analyses can be described as follows: When O_2_ was present in the flue gas, SO_2_ was mainly removed by a combined effect of physisorption and chemisorption on the FZC sorbent. The catalytic oxidation effect of active sites promoted the oxidation of SO_2_ to SO_3_. SO_3_ further reacted with metal oxide to form a sulfate linked to iron, zinc, and copper without desorption to the gas phase. Metal sulfides were also formed during desulfurization. Steam in the flue gas enhanced the desulfurization performance of the FZC sorbent by reacting with SO_3_ to form H_2_SO_4_ in the porous structure of the sorbent [23]. At longer residence times, the metal sulfides, metal sulfates, and H_2_SO_4_ precipitated in the porous structure of the sorbent blocked the sorbent pores, leading to sulfidation and deactivation of the sorbent. The possible stoichiometric reactions during the desulfurization of flue gas over the FZC sorbent can be described as follows:

SO_2_ + C-[O] →SO*_3_ (6)

SO_2_ + O_2_ + C →SO*_3_ (7)

MOX + SO*_3_ →MSO_4_ (8)

SO*_3_ + M + C → MS + CO (9)

MSO_4_ + C → MS + CO (10)

SO_2_ + 1/2 O_2_ + H_2_O + C → C-H_2_SO_4_ (11)

SO*_3_: adsorptive SO_3_; C-[O]: oxygenated functional groups on surface of char; M: metal.

## 4. Conclusions

(1) Compared with lignite char, the char-supported Fe-Cu-Zn sorbents showed significantly better flue gas desulfurization performance in the temperature range of 100–300 °C. The sulfur uptake capacity of the FZC sorbent increased with temperatures up to 250 °C.
(2) During desulfurization, the catalytic oxidation of SO_2_ with oxygen functional groups on the char surface and oxygen in the flue gas was observed. Active metal oxides promoted the catalytic oxidation of SO_2_ to SO_3_, enhancing the desulfurization of the flue gas. The adsorbed SO_3_ further reacted with metal oxides to form metal sulfates. Metal sulfides were also formed as a product of desulfurization.
(3) XRD results confirmed that the active components of zinc ferrite (ZnFe_2_O_4_), copper ferrite (CuFe_2_O_4_), Fe_3_O_4_, ZnO, and Cu were converted into metal sulfates and metal sulfides as a result of SO_2_ removal by the FZC sorbent.
(4) The presence of steam in the flue gas enhanced the desulfurization by reacting with adsorbed SO_3_ to form H_2_SO_4_, which was then retained in the porous structure of the sorbents.

## Figures and Tables

**Table 1:** Proximate and ultimate analyses of raw lignite.

Proximate Analysis, wt./% (ad)				Ultimate Analysis, wt./% (ad)				
M	A	V	FC	C	H	O *	N	S
19.96	4.03	30.89	45.12	58.09	3.39	13.9	0.46	0.17

ad: air-dried basis; *: calculated by difference; M_ad_: moisture content on an air-dried basis; A_ad_: ash content on an air-dried basis; V_ad_: volatile matter content on an air-dried basis; FC_ad_: fixed carbon content. C: Carbon; H: Hydrogen; O: Oxygen; N: Nitrogen; S: Sulfur.

**Table 2:** Experimental conditions used in this study.

Items	Desulphurization
Temperature (°C)	100–300
Space velocity (h^−1^)	1500
Gas composition (ppm)	SO_2_: 2000
Gas composition (vol. %)	O_2_: 5
	Steam: 10
	N_2_: balance

**Table 3:** Physical parameters of lignite char and FZC sorbent.

Samples	BET Surface Area (m^2^/g)	Pore Volume (cm^3^/g)	Pore Size (nm)
Lignite char	62.59	0.037	2.41
FZC	360.71	0.23	1.18

**Table 4:** Assignments of FTIR bands.

Wavenumber (cm^−1^)	Assignments
3450	O–H, in hydroxyl groups
2923	C–H, in methyl and methylene groups
2362	C≡C
1630	metal sulfide and metal sulfate characteristic adsorption band
1590	C=O, carboxyl-carbonates
1220, 1100	C–O, in ethers
1056	–SO_3_, symmetric stretching vibration
1210, 1040	SO_4_^2−^, characteristic adsorption band
600,480	* MOx, MSO_4_, and MS characteristic adsorption band

* MOx—metal oxide; MSO_4_—metal sulfate; MS—metal sulfide.

## Data Availability

The data presented in this study are available on request from the corresponding author due to privacy.
